# Facilitators and barriers for implementation of health programmes with Māori communities

**DOI:** 10.1186/s43058-024-00567-y

**Published:** 2024-03-18

**Authors:** John Oetzel, Renei Ngawati, Darrio Penetito-Hemara, Tori Te Puke, Akarere Henry, Sulita Povaru-Bourne, Dianne Sika-Paotonu

**Affiliations:** 1 University of Waikato, Private Bag 3105, Hamilton, 3240 New Zealand; 2 Te Hotu Manawa Māori trading as Toi Tangata, 9 Kalmia Street, Auckland, 1051 New Zealand; 3 South Waikato Pacific Islands Community Services Trust, 1 Maraetai Lane, Tokoroa, 3420 New Zealand; 4 University of Otago, Wellington, 23A Mein Street, Wellington, 6242 New Zealand

**Keywords:** Māori, Implementation barriers, Implementation facilitators, Health equity, Indigenous

## Abstract

**Background:**

Addressing health inequities that Māori (Indigenous peoples) communities face in New Zealand is a key aim of researchers and practitioners. However, there is limited understanding of the implementation processes and outcomes of health programmes for addressing these inequities. The aim of this study was twofold: (a) to identify correlates of implementation outcomes and (b) to identify facilitators and barriers to implementation effectiveness.

**Methods:**

The study involved a concurrent mixed method approach. Through an online survey, 79 participants with experience in implementing a health programme with a Māori community identified outcomes and processes of the programme. Additionally, nine Māori community providers shared their perceptions and experience of facilitators and barriers to implementation effectiveness through an in-depth interview. The quantitative and qualitative findings were integrated to address the aims of the study.

**Results:**

For the first aim, we identified two key outcomes: overall health impacts and sustainability. Three of the variables had significant and positive bivariate correlations with health impacts: cultural alignment, community engagement, and individual skills. The only significant correlate of sustainability was evidence-based. For the second aim, participants described four facilitators (leadership, whanaungatanga [relationships], sharing information, digestible information) and four barriers (system constraints, lack of funding, cultural constraints, lack of engagement) to effective implementation.

**Conclusion:**

Overall, leadership, aligning culture, and building on whanaungatanga, while getting financial resources and systems support, are the core elements to supporting implementation efforts in Māori communities.

**Supplementary Information:**

The online version contains supplementary material available at 10.1186/s43058-024-00567-y.

Contributions to the literature

- Provides an Indigenous lens to facilitators and barriers within the Consolidated Framework for Implementation Research
- Illustrates the importance of cultural alignment and community engagement for implementation of health services and programmes in Indigenous communities
- Offers insights to implementation practice and policy within a changing health system

## Background

Māori (Indigenous peoples) experience more health inequities relative to non-Māori populations in Aotearoa New Zealand including living shorter lives and dying from preventable diseases [1, 2]. The life expectancy for Māori is 7 years less than for other New Zealanders and the major causes of death are preventable and treatable [3]. These poor health outcomes are explained by differential exposure to determinants of health such as socioeconomic differences and level of deprivation, structural racism, colonial history, inequitable access to health and social systems, and not following the principles of Te Tiriti o Waitangi (Treaty of Waitangi) [3–5]. Te Tiriti is the founding document of Aotearoa New Zealand and is a disputed document that has different versions in English and Māori, but guaranteed Māori rights. In modern times, Te Tiriti is grounded in five principles around healthcare: (a) Recognition and protection of tino rangatiratanga (self-determination); (b) Equity—equal access to health care and equitable outcomes; (c) Active protection—governmental protection to ensure the first two principles are met; (d) Partnership—government sharing decision-making and governance with Māori; and (e) Options—providing options for services that are grounded in Te Ao Māori (Māori worldview) [1].

Despite these principles, the best health programmes (preferred as opposed to interventions because intervention has a negative connotation in some Māori communities because it implies a problem needing to be fixed from the outside) and services are not equally translated to Māori communities [1, 3]. When these programmes are options for Māori communities, the implementation of them may not acknowledge self-determination, may not be culturally appropriate, or may not be timely [6–10]. Access to the latest research and innovations can help address health inequities [11, 12] and there are health programmes and services that show promise for addressing the preventable and treatable diseases for Māori communities [13–16]. While these programmes have shown to have efficacy, most of them have not considered larger issues of implementation science and effectiveness.

This study seeks to advance understanding of facilitators and barriers to implementation effectiveness in Indigenous communities. In addition to identifying how implementation barriers and facilitators are associated with implementation outcomes (aim one), this study seeks to understand the perspective of community providers who are most often tasked with implementing health programmes for their communities (aim 2).

Two implementation science frameworks guide this study. The Consolidated Framework for Implementation Research (CFIR) is a prominent model in the literature. CFIR provides a comprehensive framework by integrating 19 different implementation science models and theories [17, 18]. CFIR is comprised of five elements: intervention (what is being implemented), process (how it is implemented), inner setting (team and organisation implementing the intervention), outer setting (social and political factors), and individuals (those who do the implementation) [17]. The He Pikinga Waiora (Enhancing Wellbeing) Implementation Framework provides a specific framework for Indigenous communities [19]. HPW is centred around Māori epistemologies and mātauranga (knowledge) along with four supporting elements of the framework: community engagement, culture centredness, systems thinking, and integrated knowledge translation. These elements focus on understanding a holistic perspective of the community and the system for the implementation project, while also having shared partnership and decision making with communities and community organisations to ensure the programme has strong alignment with the cultural aspects of the community [19].

Both frameworks provide useful guidance to implementation processes as they help to identify facilitators and barriers to implementation effectiveness. However, the empirical literature in Māori communities is limited with a few exceptions [20–22]. These studies emphasise the importance of a co-design process, community engagement, and providing a flexible structure. Barriers include lacking funding and having organisational constraints related to staffing and research capacity. In the current study, the two frameworks provided the direction for the data collection: (a) the survey was based on the elements of the frameworks and (b) the interviews including probing questions related to the elements.

## Methods

### Research design

A concurrent mixed methods research design was used for this study [23]. Qualitative data were collected via in-depth interviews and quantitative data were collected through an online cross-sectional survey. These data were integrated to explore barriers and facilitators when implementing health programmes in Māori communities. This study was part of a larger project and supported the Healthier Lives Implementation Network, which is a network of researchers and community providers and researchers in Aotearoa New Zealand to facilitate the translation of health research programmes into practice using co-design/participatory approaches [24].

### Sampling

The sampling frame were researchers, health system representatives, and community providers who implemented a health programme in a Māori community. Community providers are holistic health and social service providers who deliver services from a Māori worldview and primarily serve Māori communities. We did not have a specific sampling frame from which to recruit so we used a purposive sampling approach of three sources. First, we invited researchers affiliated with the Healthier Lives National Science Challenge to complete the survey (*n* = 343). Second, we invited members of our implementation network including people who participated in the co-design process for the development of the network (*n* = 120). We also asked invitees to share the survey with their own networks. Finally, we invited people who are affiliated with the community providers in the network and have done implementation of health programmes to participate in the interviews. We conducted interviews until theoretical saturation was reached.

### Measures

In the survey, we asked participants to recall their most recent implementation and requested descriptive information. We then asked questions about six categories using the CFIR and HPW frameworks [17, 19] as guides on key components about implementation process and effectiveness: outcomes (6 items), programme (CFIR; 7 items), process (CFIR and HPW; 7 items), organisation (CFIR; 7 items), community engagement (HPW; 4 items), and individuals (CFIR; 5 items). The measures were based on a 4-point scale from strongly disagree (1) to strongly agree (4). The outcome items included a not applicable response in case that specific outcome was not relevant to the project. The measures were previously used (with minor adaptations) and validated in a New Zealand sample [20] based on an international review of implementation measures of facilitators and barriers [25].

A semi-structured interview protocol was employed with questions based on previous research [21, 26]. We used an interpretive approached design to explore participants’ perspectives and experiences. We had questions about capacity, current needs, implementation readiness, factors that would enable and hinder implementation, advantages and disadvantages of using another organisation’s programme, and resources needed for implementation. Additional file 1 includes the survey questions and interview protocol.

### Procedures

Ethics approval was provided by the University of Waikato’s Human Research Ethics Committee (Health2022#45). Qualtrics was used to administer the survey. The survey link was sent to participants via email that had been provided to us from participants directly or through the Healthier Lives National Science Challenge own database. Community researchers conducted the interviews over zoom. Participants returned an informed consent via email and provided oral consent and permission to record the interviews. The recordings were transcribed and shared with participants for review. A $50 koha (gift; petrol or grocery voucher) for participating in either the survey or interview was offered. All documents and interviews were conducted in English to facilitate consistency.

### Data analysis

Prior to conducting the primary data analysis of the survey data, we reviewed missing values and replaced them with series mean after establishing they were missing at random (only 0.5% missing data; some participants (*n* = 10) did not complete entire sections, and these were removed from further analysis). Exploratory factor analysis was used to identify subscales within the six categories; Cronbach’s alpha for the (sub)scales were used to establish reliability. Descriptive statistics included means, standard deviations, and confidence intervals for continuous data and frequencies for categorical data. Finally, bivariate correlations and multivariate regression models were used to identify the correlates with implementation outcomes. Analysis was undertaken with SPSS 30 [27].

Thematic analysis was used to analyse the interviews [28]. The thematic analysis was conducted by one Māori researcher (RN) and one non-Māori team member (JO). They worked together to identify initial codes and then RN completed the open coding process. Both analysts independently moved the open codes to themes and then shared their results with each other. Minor discrepancies emerged and these were resolved through discussion. Themes were shared with network members as a validity check.

## Results

### Aim 1: correlates of implementation outcomes

There were 119 respondents to the questionnaire. As we did not have a clear number of total invitations, we are unable to provide a direct response rate. Of this total, 19 people indicated they had never been part of an implementation of a health programme and thus they were not asked to complete the survey. Of the 100 remaining participants, 39 indicated they had part of an implementation project with a Māori community, 18 with a Pacific community, 40 with both, and 3 with a different community. However, 15 of these participants did not move past this initial question resulting a final sample of 85. Table 1 presents a summary of the demographic characteristics of the study sample and the programmes they recalled.

**Table 1:** Demographic characteristics of participants

Characteristic	Attribute	Frequency
Gender	Female/Wāhine	41
	Male /Tāne	20
Ethnicity	Māori	36
	Pacific	12
	European	13
Job position	Management/Leadership	13
	Kaimahi/Community Worker	20
	Researcher	18
	Clinical staff	8
Health issue of programme	Diabetes	20
	Lifestyle	45
	Cardiovascular disease	17
	Cancer	9
	Health Education	34
	Health Promotion	37
	Quality of life improvement	28
	System change	24
	Mobile communication tools	8
	Other	20
Role within the programme	Deliverer/Care provider	38
	Evaluator	11
	Co-Creator	29
	Management/Supervisor	19
	Cultural Advisor	12
	Advisory board member	4
	Principal investigator	8
	Funder	7
	Other	9
Origin of the programme	Ministry of Health	15
	Other Health authority (e.g. Te Whatu Ora)	25
	Developed by researchers	12
	Developed by your community and community provider	24
	Developed by your community and researchers	19
	Developed by a different community provider who shared it	7
	An overseas programme	1
	Other	12

Prior to addressing the primary research questions, the items for the six main implementation categories were subjected to factor analysis (see Additional file 2 for results). Table 2 provides the descriptive statistics for the resulting variables including a correlation matrix and Cronbach’s alphas for the scales. This table indicates that most of the variables were at, or slightly to moderately above, agree on the scale indicating positive viewpoints about the implementation variables and outcomes. Variables that were about a third of a point higher than the agree point included cultural alignment, health impacts, community engagement, and management. The lowest factors were evidence-based and sustainability which were at or lower than the agree point.

**Table 2:** Descriptive statistic and correlations

Variable	M	SD	95% CI	Outcome	Cultural fit	Co-design process	Organisation	Team	Community	Individual	Sustainability	Evidence	Evaluation
Outcome	3.35	.46	3.25, 3.45	.84									
Cultural Fit	3.42	.50	3.31, 3.53	.494^**^	.89								
Co-design process	3.17	.51	3.05, 3.28	.238^*^	.522^**^	.87							
Organisation	3.33	.53	3.21, 3.45	.222	.398^**^	.558^**^	.83						
Team	3.23	.62	3.09, 3.38	.213	.372^**^	.462^**^	.551^**^	.89					
Community	3.28	.55	3.16, 3.41	.316^**^	.643^**^	.606^**^	.493^**^	.490^**^	.86				
Individual	3.37	.49	3.25, 3.48	.431^**^	.562^**^	.571^**^	.537^**^	.698^**^	.406^**^	.90			
Sustainability	3.00	.77	2.82, 3.17	.151	.206	.201	.157	.107	.146	.122	-		
Evidence	2.94	.77	2.77, 3.11	.071	.191	.214	.252^*^	.316^**^	.129	.325^**^	.226^*^	-	
Evaluation	3.17	.57	3.05, 3.29	.208	.130	.308^**^	.230^*^	.292^*^	.180	.261^*^	.079	.023	-
Funding Constraint	2.45	.79	2.27, 2.63	− .150	− .284^*^	− .301^**^	− .225	− .174	− .254^*^	− .138	− .132	.023	− .010

Values on the diagonal are Cronbach’s alpha; sustainability, evidence, evaluation, and funding constraint were single items

*p<.05

**p<.01

For the first aim, we only included those who had experience with implementation in a Māori community (*N* = 79). The multiple regression model of implementation variables on health impacts was statistically significant, F(2,56) = 12.54, *p* < 0.001, adj R^2^ = 0.29. While three of the variables had significant and positive bivariate correlations with health impacts (cultural alignment, community engagement, and individual skills), the regression model found that cultural alignment (B = 0.26, SE = 0.10, Beta = 0.34, *p* < 0.05) and individual skills (B = 0.23, SE = 0.10, Beta = 0.30, *p* < 0.05) were statistically significant correlates of health impacts. The multiple regression model of implementation variables for sustainability was statistically significant, F(1,57) = 4.40, *p* < 0.05, adj *R*^2^ = 0.06. The only significant correlate was evidence-based (B = 0.26, SE = 0.12, Beta = 0.27, *p* < 0.05).

### Aim 2: implementation barriers and facilitators

There were nine interviewees and all were Māori with eight females and one male; 11 participants were invited, but two decided not to participate due to time constraints. The participants represented community providers and Māori public health agencies who worked closely with community providers. There were three CEOs or general managers, four kaimahi (workers), one cultural advisor, and one consultant. Four worked in rural settings and five in urban areas with some rural outreach. Table 3 presents the themes and an exemplar quote. Additional file 3 provides a detailed description and multiple quotes for each theme.

**Table 3:** Exemplar quotes and description of key themes

Theme	Description	Quote
*Facilitators*		
Leadership	Leading projects to ensure the community needs are meet in the implementation	Or even if we’re not doing a service, even if we do a community approach if it was around kai and nutrition and stuff, what are the factors that lever to leverage changing collectives even if it is organic in community led. (M1)
Whanaungatanga	Building trusting relationships to support effective implementation and learning from others	I think there’s a few things I’d probably in the first instance, making sure that the – I don’t know if it’s a resource or a tool but one of the enablers is whanaungatanga, like the relationship with the whānau (extended family), the community, the activator’s probably the primary resource because if you don’t have that connection, you’re not getting in. (M2)
Sharing information	Sharing existing programmes and experiences with others to help everyone benefit	To have a resource readily available to see what others are doing, as well as to make it like some sort of networking tool as well so that organisations or whoever are keeping up-to-date on what they’re doing out in the community and stuff like that. (M5)
Digestible Information	Providing information in a way that makes sense for providers and communities helps to support implementation	All of our resources come back-to-back in English and te reo, so that whānau can have both. They can translate it for themselves. They can see what each thing says. We always make sure that our resources are real simple and not convoluted messaging – just really straight forward but we’ll talk to it. (M4)
*Barriers*		
System constraints	Health system privileges researcher perspectives and medical solutions rather than focusing on social determinants and community perspectives	GPs are trained to treat a problem, to fix it, to put something on it, to band aid it up and fix it. But what we know is that’s not sustainable for whānau. That’s literally band aiding it. It's not solving the issue. It's a bit like when someone goes to MSD and they’re short of kai so we’ll give them a food pack. Cool, you have fixed the problem for today, but you actually haven’t addressed the issue that this whānau is not able to sustainably feed themselves.(M4)
Lack of funding	Not having fundings limits the ability of providers to effectively develop and implement programmes	Resources, kaimahi [workers], a wāhi a place to run it. Being able to pay the kaimahi properly. Value their Kaupapa [ideas, approach]. Having access to kaimirimiri [massage therapist] so it’s a lot of networking, … We learn through wānanga [extended workshops] while sitting and talking and showing our pūrākau [stories], so creating a safe space that whānau can sit in and be able to do that is really crucial to help the information and the kōrero [conversation] flow. (M3)
Cultural constraints	Not understanding the different needs, beliefs and language barriers within cultural communities limits programme effectiveness	It needs to have consistency in values, tikanga and all those sort of things. If we were to combine the two for instance, when we had to do all the Covid vaccinations and that, we had limited space. They wanted to use one of the rooms that had a kitchen in it. I said, “We shouldn’t really be mixing kai and blood and skin samples,” and all sorts of things they were doing. They hadn’t thought of that. (M8)
Lack of engagement with communities	Not having connections and relationships with the community limits effectiveness of implementation	In our knowledge, nobody has ever come to the iwi (tribe) to ask how the health services are being rolled out in our area. Nobody has come to ask us that. We think that is a failure of any system. (M9)

There were four themes related to facilitators: leadership, whanaungatanga (relationships), sharing information, and digestible information. Leadership focused on an organisation leading on behalf of the community and also having a community-led project. Whanaungatanga was about building networks as a relational and cultural requirement to establish trust before implementing a programme. Sharing information was the importance of collaboration and specifically in sharing information with others and learning from others. Digestible information was about having information that fits a Māori viewpoint and is relevant to community members.

There were four themes related to barriers: system constraints, lack of funding, cultural constraints, and limited engagement. System constraints focused on how funding bodies limited what providers could do. Lack of funding limited the resources and capacity in the workforce which limited implementation possibilities. Cultural constraints acknowledged that mainstream organisations and researchers did not follow a Māori cultural perspective or tikanga (cultural protocols) which negatively impacted implementation. Limited engagement was also recognised as occurring with mainstream organisations and was reflected as not engaging in a participatory or co-design manner.

### Integration of findings

The mixed methods findings illustrate how CFIR and HPW provide insights on the facilitators and barriers. Facilitators included aspects of the inner setting (leadership), individuals (individual skills), outer setting (sharing information), the programme (digestible information), and the process (relationships, community engagement, and cultural alignment). The three process elements are closely aligned with the HPW framework, while the other aspects are consistent with CFIR. The survey data emphasised process and individual skills, while the interviews focused on the settings, programme, and process. The barriers came only from the interview data and included aspects of the outer setting (system constraints and lack of funding) and process (cultural constraints and limited engagement). The process items reflect HPW and the outer setting aligns with CFIR.

## Discussion

Several of the facilitators are consistent with prior research on implementation process in Māori communities and several provide new insights. Key foundational facilitators from this study, and consistent with the literature, are tino rangatiratanga (self-determination), leadership, and whanaungatanga (relationships) [21, 22]. These values and practices are core to Te Ao Māori (Māori worldview). Similarly, the importance of cultural alignment and community engagement are factors that ensure the relevance of the programme to the local community and provide cultural safety [19, 21, 22, 29]. In contrast to a prior study which examined general health professionals [20], the current study focused on participants from community providers or who partner with community providers. These providers are grounded in Te Ao Māori and hence why cultural alignment may be a stronger facilitator than the other elements. Additional novel factors found in this study are the importance of digestible information, individual skills, and evidence base for sustainability.

Barriers from this current study are largely consistent with the existing literature. Lack of funding and system constraints have been previously identified [21]. Not having sufficient funding limits the capacity of community providers; several participants mentioned they had unmet needs in their community because they simply did not have the resources to address all their community members. Further, a system that does not work to support the implementation efforts of the community provider was a key barrier. One element noted by participants is that the system constraints impede them from developing the evidence-base need to support their work. Additional barriers were lack of engagement and lack of cultural alignment; these are opposites to the facilitators, but are consistent with prior research that demonstrates that facilitators and barriers are not fixed and can be enablers or barriers depending on how they are applied [30].

There are several implications for research and practice. The current study found facilitators and barriers can be categorised within the five aspects of the CFIR: individuals, inner setting, outer setting, programme, and process [17, 18]. Further, the findings support research on Indigenous perspectives of facilitators and barriers for implementation effectiveness [19, 30]. The findings of this study have important implications for policy and practice, especially within the context of the principles of Te Tiriti (Treaty). The facilitators of effective implementation are consistent with the principles of tino rangatiratanga (self-determination), equity, active protection, partnership, and options grounded in Te Ao Māori (Māori worldview) [1]. Thus, the health system and funding streams should seek policies and strategies that require partnership, community engagement, and culturally resonant options for services and programmes [19].

This study does have several limitations. A relatively small and non-random sample does not allow us to generalise to the larger population. The sample size does have limitations in determining the factor structure and predictor coefficients although we did meet minimum requirements [31–33]. Further, we were not able to identify a response rate. Additionally, the interview participants focused on general implementation points which introduce additional heterogeneity. Nonetheless, consistency with the extant literature and guiding principles of Te Tiriti (Treaty) reinforce the importance of these facilitators and barriers. Finally, we did not explore the specific elements of CFIR or HPW in actual implementation projects; rather we relied on perceptual and recall data to identify facilitators and barriers.

## Conclusions

This study contributes to the implementation science literature for Indigenous communities. Specifically, it identified that rangatiratanga (leadership and self-determination), cultural alignment, and building on relationships with other organisations and the community, while getting financial resources and systems support, are the core elements to support implementation efforts. This study identifies culturally specific elements to implementation science that are important to address health equity issues in New Zealand. While developing new programmes and services that address key health issues are needed, if the programmes and services are not implemented effectively, they will fail to achieve the desired objectives. Māori community providers are well situated to share their experiences on facilitators and barriers of implementation and hopefully the health system will seek their guidance and partner with them to meet the needs of Māori communities.

### Supplementary Information

**Additional file 1.** Survey items and interview protocol.

**Additional file 2.** Factor analysis of implementation items.

**Additional file 3.** Detailed thematic analysis.

## Data Availability

The datasets used and/or analysed during the current study are available from the corresponding author on reasonable request.

## Glossary

**Te Reo Māori**: **Approximate English Translation**
*Aotearoa*: New Zealand
*He Pikinga Waiora*: Enhancing Wellbeing
*iwi*: tribe
*kaimahi*: workers
*kaimirimiri*: traditional massage therapist
*kaupapa*: idea, programme
*koha*: gift
*kōrero *: talk, conversation
*Māori*: Indigenous people of New Zealand
*mātauranga*: knowledge
*pūrākau*: stories
*Te Ao Māori*: Māori worldview
*te reo*: language
*Te Tiriti o Waitangi*: Treaty of Waitangi
*tikanga*: cultural protocols
*tino rangatiratanga*: self-determination
*wānanga*: extended workshops
*whānau*: extended family
*whanaungatanga*: relationships
