# Supplementary material for: Facilitators and barriers for implementation of health programmes with Māori communities
Source: Implement Sci Commun. 2024 Mar 18;5:26. doi: 10.1186/s43058-024-00567-y (PMC10946171; doi:10.1186/s43058-024-00567-y)
Supplement: Supplementary file 1 — Additional file 1. Survey items and interview protocol. [file 43058_2024_567_MOESM1_ESM.docx]

**Supplemental File One: Survey Items and Interview Protocol**

***Facilitators and barriers for implementation survey***

Have you been a part of or supervised a project implementing a health programme with Māori or Pacific populations? (If you were involved in creating, managing, funding or sourcing funding, delivering, researching, or evaluating say yes). Select only one response

Yes with a Māori community

Yes with a Pacific community

Yes with both Māori and Pacific communities

No, but I’ve implemented health programmes in other communities

No

Please think about the most recent project that you have helped implement and that you have some sense of how effective it is or was. It can be something that worked well or didn’t work so well.

Please select what the programme was about. Tick all that apply:

1. Diabetes b) Lifestyle c) Cardiovascular Disease d) Cancer

e) Health Education f) Health Promotion g) Quality of Life Improvement h) Systems Change i) Mobile communication tools for health (apps) j) Other: ­ _______________________

Please share what your role(s) was with this implementation. Tick all that apply:

1. Deliverer/care provider b) Evaluator c) Co-creator d) Manager/Supervisor e) Cultural advisor f) Advisory Board Member g) Principal Investigator h) Funder i) Other: ­­­­­­­­­­­­­­________________________________

Please tell us where the programme came from (tick all that apply)

1. Ministry of Health b) Other health authority (such as Te Whatu Ora) c) Developed by researchers d) Developed by your community or community provider e) Co-developed by researchers and the community provider f) Developed by a different community provider who shared it with you g) An overseas programme h) Other:___________________________

*Outcomes*

This section is based on the outcomes of the most recent health programme you were involved in. Based on your experience, rate your level of agreement that the programme resulted in each of the following:

| Item |  | Strongly agree | Agree | Disagree | Strongly Disagree | Not Applicable (not a goal of the programme) |
| --- | --- | --- | --- | --- | --- | --- |
| 1 | Better co-ordination between health providers and community groups |  |  |  |  |  |
| 2 | Improved the health outcomes of those who participated |  |  |  |  |  |
| 3 | Improve the health behaviours of people who participated |  |  |  |  |  |
| 4 | The programme has continued after the initial funding period |  |  |  |  |  |
| 5 | The programme was well received by the community |  |  |  |  |  |
| 6 | Improved the health of the community |  |  |  |  |  |

*Programme*

This section considers the characteristics of the programme; it focusses on WHAT was implemented. Based on your experience with the most recent programme implemented, please indicate your level of agreement that each of the following occurred during the implementation of the programme.

| Item |  | Strongly agree | Agree | Disagree | Strongly Disagree |
| --- | --- | --- | --- | --- | --- |
| 1 | The programme had clear structure and guidelines that were shared with those implementing the programme. |  |  |  |  |
| 2 | The programme was consistent with the customs and cultural practices of the community. |  |  |  |  |
| 3 | There was evidence supporting the effectiveness of the programme from national or international studies prior to implementing the programme. |  |  |  |  |
| 4 | The programme was adapted to fit the needs of the community |  |  |  |  |
| 5 | The community or community provider was ready and wanting to implement the programme |  |  |  |  |
| 6 | The programme was consistent with the values and principles of the community |  |  |  |  |
| 7 | The original creators of the programme shared their experience to facilitate the implementation of the programme. |  |  |  |  |

*Process*

This section addresses the processes of implementing a health programme; it focusses on HOW the programme is created and implemented. Based on your experience with the most recent programme implemented, please indicate your level of agreement that each of the following occurred during the implementation of the programme.

| Item |  | Strongly agree | Agree | Disagree | Strongly Disagree |
| --- | --- | --- | --- | --- | --- |
| 1 | The implementation team evaluated what they do well and how to improve on collaboration |  |  |  |  |
| 2 | The programme was the result of shared decision making amongst community and other partners. |  |  |  |  |
| 3 | At meetings with external stakeholders, the implementation team worked collaboratively with all members |  |  |  |  |
| 4 | Relevant external stakeholders were included in the implementation of the programme |  |  |  |  |
| 5 | Participating in implementing the health programme helped the team to see the complexity of the health issue |  |  |  |  |
| 6 | There was a strong partnership between the community, the implementation team, and other stakeholders. |  |  |  |  |
| 7 | The funder supporting the programme placed a lot of constraints during implementation. |  |  |  |  |

*Organisation*

This section is based on your views of the support of an organisation that is responsible for implementing the health programme; this section focusses on WHO is involved in implementing the programme. Based on your experience with the most recent programme implemented, please indicate your level of agreement that each of the following occurred during the implementation of the programme.

| Item |  | Strongly agree | Agree | Disagree | Strongly Disagree |
| --- | --- | --- | --- | --- | --- |
| 1 | The programme was important to the management of the organisation responsible for implementation |  |  |  |  |
| 2 | Management supported the programme actively |  |  |  |  |
| 3 | The board/managers provided stewardship of the activities of the project team |  |  |  |  |
| 4 | The division of tasks in the implementation team was perfectly clear |  |  |  |  |
| 5 | Everyone in the implementation team did what they needed to do |  |  |  |  |
| 6 | There was good communication and coordination in the implementation team. |  |  |  |  |
| 7 | The organisation had sufficient capacity to carry out the implementation |  |  |  |  |

*Community*

This section is based on your views of the community that received the programme; this section focusses on WHO is receiving the programme. Based on your experience with the most recent programme implemented, please indicate your level of agreement that each of the following occurred during the implementation of the programme.

| Item |  | Strongly agree | Agree | Disagree | Strongly Disagree |
| --- | --- | --- | --- | --- | --- |
| 1 | The community was engaged with prior to the implementation |  |  |  |  |
| 2 | The community identified the programme as addressing a key health need |  |  |  |  |
| 3 | The community was committed to the implementation of the programme |  |  |  |  |
| 4 | The community worked collaboratively with the organisation to implement the programme |  |  |  |  |

*Individual*

This section considers the role of individual team members during the implementation of the health programme. Based on your experience with the most recent programme implemented, please indicate your level of agreement that each of the following occurred during the implementation of the programme.

| Item |  | Strongly agree | Agree | Disagree | Strongly Disagree |
| --- | --- | --- | --- | --- | --- |
| 1 | Individuals in the team were able to contribute new ideas to the implementation rather than just follow established protocols |  |  |  |  |
| 2 | Individuals felt adequate in their role to fufill the implementation of the programme |  |  |  |  |
| 3 | Individuals were aware of different cultural perspectives during the implementation of the programme |  |  |  |  |
| 4 | Individuals were able to overcome barriers during the implementation process. |  |  |  |  |
| 5 | Individuals who implemented the health programme were confident in their ability to do so |  |  |  |  |

*Demographics*

The final section are questions on your basic background information, all of which will be kept confidential and will not require you to reveal your identity.

Please fill in the blanks:

1. Gender identity: _________________
2. Ethnicity: ________________
3. Job position: ___________________
4. In what sector do you work?
5. Community researcher or community provider
6. University or Crown Researcher
7. Health system (e.g., Te Whatu Ora, Te Aka Whai Ora, Ministry of Health)
8. Other:______________

***Interview Protocol***

The following questions are tentative interview/focus group questions for data collection around implementation needs barriers, etc.

1. What are your current needs around health in your organisation and communities?
2. How ready are you to take action to address these needs? Why is that?
3. What capacity do you have to implement a programme that might help you address these needs?
4. What would help you implement a new programme?
5. What constraints do you have in implementing a new programme?
6. What are the potential benefits of using an existing programme that was developed by another organisation/location?
7. What are the potential disadvantages using an existing programme that was developed by another organisation/location?
   1. What would you need to do to make sure it would work well for you?
8. Resources
   1. What resources would you like to have (from the original project) to aid its implementation?
   2. What resources from the network do you think you’d need?
9. Any other points you’d like to add?
