# Supplementary material for: Facilitators and barriers for implementation of health programmes with Māori communities
Source: Implement Sci Commun. 2024 Mar 18;5:26. doi: 10.1186/s43058-024-00567-y (PMC10946171; doi:10.1186/s43058-024-00567-y)
Supplement: Supplementary file 2 — Additional file 2. Factor analysis of implementation items. [file 43058_2024_567_MOESM2_ESM.docx]

**Supplemental File Two: Factor Analysis of Implementation Items**

We completed exploratory factor analysis using the data from all participants in the study. A principal components analysis with varimax rotation was utilized using SPSS. The sample is relatively small, but meets an oft-cited criterion of 10 participants per item for each specific analysis, ^[[1]](#endnote-1)^ although some advocate for as few as five participants per item. Another study considers the ratio of number of items, items to factors, number of factors, and communalities.^[[2]](#endnote-2)^ With the conditions in the current study, a sample size of 75-90 provides good estimates relative to the population structure (N=85 in this study). The small sample size is a limitation of this analysis and future research should replicate the factor structure of this current study.

The six outcome items resulted in two factors accounting for 70.03% of the variance. The factors included health impact (4 items; ∝=.84) and sustainability (1 item). Health impact was whether the programme had positive behavioural change or health outcomes. Sustainability was whether the programme continued beyond the initial funding period.

| **Outcome Items** | 1 | 2 |
| --- | --- | --- |
| Better co-ordination between health providers and community groups | .601 | .487 |
| Improved the health outcomes of those who participated | .878* | -.016 |
| Improve the health behaviour of people who participated | .786* | .189 |
| The programme has continued after the initial funding period. | .016 | .952* |
| The programme was well received by the community | .720* | .098 |
| Improved the health of the community | .862* | .059 |

The seven items about the programme resulted in two factor accounting for 70.81% of the variance. These factors were cultural fit (4 items; ∝=.89) and evidence-based (1 item). Cultural fit refers to the fit or alignment of the programme to the community’s culture; evidence-based addressed whether there was national or international evidence supporting the programme.

| **Programme Items** | 1 | 2 |
| --- | --- | --- |
| The programme had clear structure and guidelines that were shared with those implementing the programme | .457 | .535 |
| The programme was consistent with the customs and cultural practices of the community | .845* | .078 |
| There was evidence supporting the effectiveness of the programme from national or international studies prior to implementing the programme | -.063 | .861* |
| The programme was adapted to fit the needs of the community | .899* | .077 |
| The community or community provider was ready and wanting to implement the programme | .732* | .369 |
| The programme was consistent with the values and principles of the community | .875* | .256 |
| The original creators of the programme shared their experience to facilitate the implementation of the programme | .477 | .671 |

The seven items for process items resulted in two factors accounting for 63.93% of the variance. The first factor was labelled co-design process (5 items; ∝=.87), while the second was not named due to a low alpha (2 items; ∝=-.01). These items were left in the analysis as single items.

| **Process Items** | 1 | 2 |
| --- | --- | --- |
| The implementation team evaluated what they do well and how to improve on collaboration | .363 | .753* |
| The programme was the result of shared decision making amongst community and other partners | .703* | -.004 |
| At meetings with external stakeholders, the implementation team worked collaboratively with all members | .860* | -.005 |
| Relevant external stakeholders were included in the implementation of the programme | .841* | -.034 |
| Participating in implementing the health programme helped the team to see the complexity of the health issue | .759* | .141 |
| There was a strong partnership between the community, the implementation team, and other stakeholders | .814* | -.055 |
| The funder supporting the programme placed a lot of constraints during the implementation | -.404 | .641* |

The factor analysis of the seven organisational items resulted in two factors accounting for 75.66% of the variance: teamwork (4 items; ∝=.89), and management (3 items; ∝=.83). Teamwork references the degree of effective coordination amongst members of the implementation team. Management included the level of support and commitment from management and the organisation’s board.

| **Organisational Items** | 1 | 2 |
| --- | --- | --- |
| The programme was important to the management of the organisation responsible for implementation | .177 | .906* |
| Management supported the programme actively | .223 | .891* |
| The board/managers provided stewardship of the activities of the project team | .348 | .680* |
| The division of tasks in the implementation team was perfectly clear | .802* | .309 |
| Everyone in the implementation team did what they needed to do | .869* | .266 |
| There was good communication and coordination in the implementation team | .817* | .328 |
| The organisation had sufficient capacity to carry out the implementation | .817* | .108 |

The four community items were clustered in one factor accounting for 70.16% of the variance (∝=.86). Finally, the 5 individual items loaded in one factor accounting for 71.59% of the variance (∝=.90). Across all of the analyses, items were removed if they did not load cleanly on the existing factors.

**References**

1. Nunnally JC: Psychometric Theory (2nd ed.). New York: McGraw Hill; 1978. [↑](#endnote-ref-1)
2. Mundfrom DJ, Shaw DG, & Ke, TL: Minimum Sample Size Recommendations for Conducting Factor Analyses, Int J Testing 2005, 5: 159-168 [↑](#endnote-ref-2)
