# Supplementary material for: Facilitators and barriers for implementation of health programmes with Māori communities
Source: Implement Sci Commun. 2024 Mar 18;5:26. doi: 10.1186/s43058-024-00567-y (PMC10946171; doi:10.1186/s43058-024-00567-y)
Supplement: Supplementary file 3 — Additional file 3. Detailed thematic analysis. [file 43058_2024_567_MOESM3_ESM.docx]

**Supplemental File Three: Detailed Thematic Analysis**

**Facilitators**

**Leadership.** The participants noted the importance of organisations leading on behalf of the community as well as the importance of having a community-led project.

“*Or even if we’re not doing a service, even if we do a community approach if it was around kai and nutrition and stuff, what are the factors that lever to leverage changing collectives even if it is organic in community led. And how do we amplify that?*” (M1, consultant)

*“We are always looking at where are the gaps, what are we missing, what are we seeing whānau [extended family] asking more questions about? For example, next week we start a gout workshop series, which we have never done before, but we are just seeing more and more of that presenting.”* (M3, kaimahi)

These providers described the leadership from community-providers to address challenges and identify how this leadership enables the providers to meet the needs of communities. They also consistently mentioned the importance of community-led initiatives, particularly through community-engaged implementation processes.

Community resilience in relation to leadership was also a point raised. Some participants included descriptions of Covid-19 responses as an example of community resilience and leadership, especially in isolated and/or rural communities.

*“One thing I have seen from our community is the resilience our community have, and that’s just not (rural community) it’s quite a lot of other small communities, you know, when you get hit with a problem and no-one comes to aid, someone somewhere is just going to stand up and do something.”* (M4, CEO)

This resilience was also related to culture as a means to be resilient in response to health and wellbeing.

“*I think the other thing that’s made a big difference to our people is the connectivity to their culture in the Māori way of looking at wellbeing. That’s a key issue for our people. They want to focus on maintaining their wellness, rather than focusing on getting rid of disease*.” (M9, CEO)

Community resilience and cultural resilience were key aspects of community-led leadership of implementation projects.

**Whanuangatanga.** Participants noted the importance of whanaungatanga or building relationships and networks to support implementation. Whanaungatanga includes networks as a relational and important cultural requirement to establish trust before implementing a programme. These relationships enable providers to offer additional resources and information to others that can help meet the needs of the community.

*“Our roadshow is whakawhanaungatanga, kapu tī [cup of tea] type of thing. I’m currently working with our local organisations, our local council bringing them altogether … we’re just going to have a real informal sit down cup of tea, kōrero [conversation], where we can discuss the needs of the community or what the current needs are in the community and what we could potentially offer to support those needs in the community.”* (M5, kaimahi)

Such relationships support providers to access other programmes that others have created and then be able to implement the programme after tailoring it to their community. This enables providers to not start from the beginning and yet they can use their own whanaungatanga to adapt the programme.

“*The fact that sometimes things have been tried elsewhere and worked very well, and that one of our strengths is being able to have a look at it and to analyse it in terms of what tweaks might need to happen, so that it would fit our people. …Implementing a programme that people are familiar with and are aware of would be easier than implementing a programme from scratch, because you’ve got to introduce it, you’ve got to socialise it and you’ve got to get people to buy into it and believe in it.”* (M9, CEO)

“*That’s the kaupapa [idea-programme] that you’d want to take. Just don’t take a little bit and then take it back to a physical wānanga where you’re kanohi kitea [face-to-face] and share that space, yeah, a little bit safer in terms of the safety of your own wairua [spirituality]*.” (M7, kaimahi)

Whanaungatanga enables trust to be established and provide a safe space for learning and sharing from others. It also facilitates the process of adapting and tailoring programmes from elsewhere to fit the local community.

**Sharing information**. Māori providers discussed the importance of collaboration and specifically in sharing information with others and learning from others. They described this as passing along mātauranga (knowledge), which is a key aspect from a Māori worldview.

“*It’s more about the mātaurangabeing handed down….So if you’re looking from a te Ao Māori perspective, it’s that handing down of mātauranga.…So that I know what I’m doing is actually pono and tika [ethical and truthful] because literally the mauri was handed to me to deliver to whānau.”* (M3, kaimahi)

*“Sharing that information allowed for other iwi [tribes] or other providers to take action in whatever way that they felt like. Some iwi were like, ‘Oh, I didn’t even think of that.’ So then they were like, ‘How can we work better with our borders?’”* (M1)

*“Yeah, absolutely we look outside of ourselves…always looking at what resources are*

*already out there developed. We don’t want to reinvent the wheel.”* (M4, CEO)

*“From my perspective and our resource here that we can give to those, everyone else has got quite a lot. I was like, you know what I can give? We’ve got statistics and data. That was me. I got a hold of our data analyst, our IT guys and was able to pull together that particular resource that others did not have, you know. But they had the other resource that I didn’t have either*.” (M7, kaimahi)

Overall, the potential benefits in collaborating with other organizations on existing programmes means learning from each other, offering resources that others do not have, understanding the insight behind the development, and being able to implement specific aspects identified.

**Digestible information**. A final facilitator for Māori providers is having digestible information for both the community and the community providers. Communicating information in a way that fits tirohanga Māori (viewpoint) and is relevant to community members can influence the level in which the community and organisation can have a relationship. Information within a programme needs to be understandable but informative to community members.

*“Just finding those places that utilise te reo [language], that utilise te Ao Māori perspective so that it’s information our whānau are going to retain. It’s not all clinical because it needs to make sense…. But it’s hitting all those different ways we learn and understand so orative, kinaesthetic, written or visual. It’s making sure that the way we teach or educate and pass information is going to hit home for whichever way they learn best.”* (M3, kaimahi)

*“She had an under 3 minutes animated clip made, that was really engaging and really beautiful and it told like a poetic Māori cartoon. Like when Rangi and Nui were separated, it broke the tapu [taboo]…. so it explained in 30 seconds, it explained the philosophical theory of change but through pictures with brief kōrero and subtitles. And then in the next 30 seconds, it explained what they did.”* (M1, consultant)

These quotes illustrate how having information that is accessible and makes sense for the target audience helps to ensure a successful implementation process and facilitates appropriate outcomes.

**Barriers**

**System constraints***.* Interview participants recognised that their ability to address health needs were commonly restricted by funding constraints and constraints in programmes. In some cases, the focus was on the system taking away programmes that were working and thus limiting what the provider can do. These system constraints limited effective implementation.

*“Like, Who thought that through? How do you put your things into the hub? ‘This is the thing we want to do,’ ‘This is the health programme we want to do.’ And it just comes up with a big “No. No. No.” In Covid there were really good examples of ‘actually there’s resistance in this community and so we approach it differently.’”* (M2, general manager)

*“Yeah, absolutely. That I think is really hard when they do discontinue stuff. Because sometimes there has been really good stuff. One example is the health promotion agency used to do ‘Player of the Day’ certificates for drink water. They’ve cut that. I’m like, sports clubs loved that and we were getting a good promotion message in there.”* (M4, CEO)

A final constraint was on where the system provided funding:

*“Yes, they [researchers] get all the mana and the money. But what we generally have is like an expert. You might have absolutely fucking expert on maternal health, doesn’t have a single qualification to their name because they’ve never been the researcher on a project they’ve been researched on.”* (M2, general manager)

This quote speaks to the expertise of community-based kaimahi who should be recognised by their skill base to contribute to research. However, the funding system favours the research system rather than the community priorities and ways of knowing.

**Lack of funding.** Another barrier is lack of funding for programme implementation, which impact resources and capacity in the workforce. Participants also noted that they were asked to do extra during the COVID pandemic without additional workforce so they are stretched and “time poor.”

*“There is no evidence base because the chicken and egg cycle year it is that you need the money to resource the research or evaluation to validate the kaupapa, but you don’t have the money. So then there is, you need the money for the kaupapa and then you need the money for the resource to evidence the kaupapa, and usually it’s one of those don’t come.”* (M2, general manager)

“*The only thing that was a little concerning was probably the pūtea [money] and funding to be able to do those sorts of things. So we managed our way around it through koha [gift] to our experts that came in to talk about it. Everything else was a collective impact*.” (M7, kaimahi)

The lack of funding also limits the number of programmes that providers can implement, but also the degree to which they can provide effective and culturally resonant evaluation of these programmes. Thus, they are not able to develop an evidence base that is grounded is te ao Māori.

**Cultural Constraints.** Not using a Māori cultural perspective and acknowledging certain barriers communities face is a key constraint for effective implementation. Tikinga (cultural protocols-practices), norms, and cultural beliefs may conflict with mainstream perspectives and lead to scepticism and even resistance towards health services. The participants explain examples of past experiences that serve as barriers to engaging with Māori communities. The also mentioned the lack of staff, particularly in the larger health system, with te reo Māori (Māori language) and Māori worldviews.

*“There’s real access barriers for whānau to whatever programme it is. There’s a cultural incompetence of whoever’s running the programme. And then there’s the ability to actually storytell what’s going on for whānau [extended family] which, for a programme to be effective, you have to be able to share that change narrative or that outcomes framework without having to fit into some kind of a reporting box.”* (M2, general manager)

*“The biggest barrier is that we don’t have people. We don’t have the mātauranga Māori. We don’t have the native speakers. We don’t have the fluent speakers. We don’t have kaumātua [elders].”* (M8, cultural advisor)

“*Finding support. Finding a Māori midwife is not easy. A lot of us don’t utilise the Pākehā [non-Māori, European] system that’s available. There isn’t a central hub place to find a Māori midwife, that kōrero through networks that we get passed around and majority of us do that purposely*.” (M3, kaimahi)

These quotes illustrate the desire to having culturally appropriate and safe practice that is consistent with Māori tikanga and worldviews.

**Limited engagement.** The final barrier identified within the interviews highlights the importance of limited community engagement and the barriers that hinder effective engagement. Participants identified the importance of engaging the community and how not having trusting relationships limits the ability to implement a programme.

*“Definitely for out here anyway. A lot of information that is provided by Ministry of Health and stuff our people out here are just like, “Nah.” Because of that they didn’t bother to build a trusting relationship with our communities. So I think that tool would be very, very helpful and more so if it’s from a te ao Māori approach too.”* (M5, kamahi)

“*One of the largest ones in Tāmaki [Auckland], I’ll be like “Yeah. Great job. Great work. You’re huge.” But you can only evidence touching 10,000 individuals and there’s 60,000 Māori in your rohe [area] that we fund you for. So what are you doing there? What can you do there? And how do you improve quality? How do you know when your quality’s not great? How do you improve it and where? And how do you communicate that to everyone else including your community? So with this research or whether it’s implementation programming and planning, I definitely don’t reckon we’ve spent enough efforts in there in the Māori space*.” (M1, consultant)

The lack of connections, engagement, and whanaungatanga is a barrier to programme implementation. Participants noted that researchers and the health system need to establish trust and engage through co-design processes when working with communities and community providers.
